# Ensuring trial conduct is consistent with trial design: assumption is the enemy of quality

**DOI:** 10.1186/s13063-019-3516-z

**Published:** 2019-07-10

**Authors:** Joanna Kelly, Barry Hounsome, Gill Lambert, Caroline Murphy

**Affiliations:** Kings College London Clinical Trials Unit, Research Management and Innovation Directorate, King’s College London, M2.06, 16 De Crespigny Park, London, SE5 8AF UK

**Keywords:** Trial design, Assumptions, Leadership, Supervision, Delegation, Protocol, Monitoring, Dataset, Reports, Analysis

## Abstract

*‘Assumptions are made and most assumptions are wrong’ (Albert Einstein)*

Clinical trial conduct must be consistent with trial design, yet conducting the trial according to plan remains a major challenge.

We discuss the importance of optimal co-applicant team formation in trial leadership, appropriate delegation of tasks and staff supervision arrangements. Finally, we discuss five standard documents which we believe require particular attention. With appropriate engagement by or with co-applicants during the preparation of these five standard documents, we believe many of the pitfalls trials commonly experience can be avoided. The risks inherent in failing to identify and address mistaken assumptions during the preparation of these documents are discussed and recommendations for best practice suggested.

## Team formation

The essence of a team is that its members form a cooperative association through a division of labour that best reflects the contribution that each can make toward the common objectives [1]. Leadership team formation is one of the first steps undertaken by an investigator conducting a clinical trial. The necessity to gather together a team is driven by the variety of tasks required to successfully deliver a trial. Many of the skills needed for a rounded trial leadership team may be unfamiliar to the investigator. If done well, this step can be hugely rewarding, laying the foundations for a successful trial.

At the apex of an optimal trial team are the lead investigator and the co-applicant statistician. Both are highly qualified by training and experience in the clinical and methodological leadership of trials. Beyond this, the wider co-applicant team provide senior specialist support that complements the expertise of the lead investigator and statistician. Responsibility to the funder for ensuring all required expertise to deliver the trial is available within the co-applicant team rests with the lead investigator.

An inexperienced lead investigator may be unaware of the breadth of skills required to deliver a trial. Advice should be sought from academic or support staff who are more experienced in trial conduct, to identify potential gaps and recommend additional co-applicants appropriate to the complexity of the particular trial. Identifying suitable senior operational co-applicants can be particularly challenging, as fixed term contracts remain common in the academic sector and many organisations have no mechanism to retain experienced senior operational staff between trials. The increase in Clinical Trials Units that can sustain operational staff on open-ended contracts is beginning to alleviate this, but more needs to be done.

In an optimal co-applicant team, staff employed or assigned to support the lead investigator in managing the trial will receive appropriate advice across all aspects of trial conduct. Each trial will have a Trial Management Group (TMG) that meets regularly to review progress and agree next steps. In an optimal trial leadership team, there will be co-applicants in the leadership team with expertise in those aspects of the trial where the lead investigator lacks personal expertise. In most trials, there will be a statistician co-applicant, because few clinical academics possess the statistical expertise themselves to design and analyse the trial. The relationship between the lead applicant and the co-applicant statistician is particularly important, as each relies heavily on the expertise on the other and so a relationship of trust is essential. Additional clinical, methodological or operational co-applicants are then invited to join the team. Co-applicants are expected to share responsibility to the funder for successful trial delivery [2]. The lead investigator will draw on the expertise of co-applicants, to inform decisions on trial conduct or confirm that proposed next steps presented by staff employed or assigned to support the trial are appropriate. Some funders, e.g. National Institute for Health Research (NIHR), permit named collaborators in addition to co-applicants and this is appropriate where specific expertise is required by the team, but the individual collaborator providing that expertise will not share responsibility for trial delivery to the funder [2].

Where a sub-optimal co-applicant team is formed, lead investigators expose themselves to a higher level of risk. If the collective team of invited co-applicants and collaborators lack the required expertise to deliver aspects of the trial or to appropriately advise and supervise the staff employed or assigned to support the trial, particular risks arise. At best, the funders themselves may identify the gap in the skill mix of co-applicants and request that additional co-applicants be invited to join the team. The grant application may be rejected if the funders are not convinced the team have the expertise to deliver, particularly if there is no co-applicant statistician. Worse, the funders may fail to identify the gaps in the team and fund the trial with a sub-optimal leadership team. In these cases, the lead applicant faces an additional challenge to deliver the trial.

Staff experienced in trial conduct who are seeking their next trial are more likely to ask for detailed information about the planned trial and to pay close attention to the entire leadership team. Trial-naïve staff are less likely to do so. Therefore, sub-optimal teams are more likely to recruit inexperienced staff. Inexperienced staff require a higher level of advice and supervision. Where the expertise is not available within the co-applicant team to support those staff, aspects of the study may stall, through lack of direction.

Safeguards are in place to compensate for sub-optimal co-applicant teams. Advice can be sought from the Trial Steering Committee (TSC) and this is the well-established mechanism by which many sub-optimal trial leadership teams manage to deliver successful trials. However, these committees usually meet once or twice a year, so the lead investigator would either need to request advice between meetings for ad hoc issues or delay the decision until the next meeting. Staff employed or assigned to the trial may become frustrated by delays in decision-making and decide to change roles. Reliance on the TSC for day-to-day management decisions is far from ideal.

The lead investigator may also seek ad hoc advice from academic colleagues, sponsor offices, pharmacists, statistics helpdesks and other local infrastructure to inform their decisions, but it cannot be assumed at grant application stage that such support will be adequate and reliance on individuals who are not co-applicants, or at least named collaborators, is to be avoided.

Embarking on any trial, particularly a large multicentre trial, without a strong mix of appropriate clinical, methodological and operational skills within the co-applicant team is to be avoided, if possible, to minimise the impact of staff turnover during the trial. Co-applicants must provide explicit clear guidance on matters relevant to the validity of the trial, particularly when the five standard documents detailed below are in development.

## Delegation of tasks

Supporting the co-applicant team, additional operational staff such as trial managers, data managers, unblinded statisticians, monitors and trial administrators may be recruited or allocated to the trial. Operational staff training and experience varies, with most educated to degree, masters or doctoral level, some with statistical or formal project management qualifications. Many lack trials methodology training beyond that learned ‘on the job’. Formal clinical trials qualifications are rare, although clinical trial courses are becoming more commonly available, some with distance learning options that permit study to be undertaken to master’s level alongside a full-time job.

Even when all aspects of a trial’s design have been specified, conducting the trial according to plan remains a major challenge [3]. When trial teams are formed, members bring with them assumptions about how trials should be conducted, based on prior education and experience. In new teams, members will form assumptions about what they perceive to be the skills, knowledge and expertise of both more senior and more junior colleagues within the team and they will make assumptions about the role expectations of themselves and others in the team. This phenomenon is not unique to trials or to academia, but the impact of such underlying assumptions reflects how effectively communications are working within the trial team.

The co-applicant team carries responsibility to the funder for trial delivery. Legally, the sponsor organisation takes ultimate responsibility for the design, management, conduct, analysis and reporting of the trial. In UK academic-led trials, the sponsor or co-sponsor organisation is typically the employing organisation of the lead investigator. The lead investigator (where they are also the chief investigator) is the individual within the sponsor organisation who takes primary responsibility for study conduct, including co-applicant team composition, identification of recruiting sites and identifying members for oversight committees. The lead investigator also employs or assigns staff to support the trial or delegates individual co-applicants to do so within their own teams. Both funders and sponsor organisations may implement governance or risk management processes to assure themselves the lead investigator decisions or recommendations are appropriate. The sponsor will ensure that collaboration agreements are in place, outlining the responsibilities of the co-applicant team and recruiting study sites. Lead investigators and co-applicants typically have significant competing clinical, teaching or other research commitments; therefore, while they retain responsibility, delegation of trial-related tasks to operational staff managed by members of the co-applicant team is common practice. This being the case, ensuring adequate oversight is important.

Examples of activities delegated by co-applicants are represented in Table 1. Maintenance of a written delegation log for tasks delegated to operational staff in the coordinating site, similar to those used in recruiting sites, is recommended. Regardless of any delegation of tasks, the co-applicants leading the trial remain responsible to the funders throughout and the lead investigator delegating the tasks remains responsible to the sponsor for oversight of those delegated. If co-applicants write protocols that lack detail, operational decisions impacting trial methodology may be sub-optimal. Reliance on individuals outside the co-applicant team to make such decisions can lead to a methodological and operational disconnect, particularly if the operational staff, staff within clinical trial units or sponsor governance teams are perceived to lead on such matters, but lack the methodological expertise to ensure their decisions do not conflict with the trial design. Equally, fortunate trial teams may find that the operational staff joining the team after funding award bring considerable additional expertise to the group. In such cases, lead investigators should be open to implementing protocol amendments if valid issues are raised, even when suggested after initial ethical approval. This is particularly important in trials where the funder requires the trial to have ethical approval in advance of grant funding, resulting in protocols being submitted to ethics ahead of grant funded staff being appointed. Without dedicated support staff employed or assigned to the trial, co-applicants commonly prepare documents for ethical review. Protocol reviews by sponsors, ethics committees and funders focus on specific clinical, scientific and methodological aspects of the study for the purpose of establishing that the study is, respectively, within an acceptable level of risk, is ethical and represents the design agreed; however, protocols can proceed successfully through these checks without the detailed content being entirely clear. There may be inconsistencies that lead to differing interpretation between staff or omission of detail that co-applicants assume is obvious. These may surface when staff employed or assigned to the trial review the protocol, when detailed processes such as data collection form design highlight protocol inconsistencies or when inconsistent processes between recruiting sites demonstrate protocol omissions.

**Table 1:** Delegation of trial tasks

Operational staff	Tasks delegated^a^
Operational trial statistician	Draft analysis plans; prepare Data Monitoring Committee reports; report to Data Monitoring Committee; conduct analyses
Trial manager	Essential document version control; seek necessary approvals; manage the intervention supply to study sites; schedule study oversight meetings; monitor study site activity; process serious adverse event reports; ethics and regulatory reporting; present to Trial Management Group
Data manager or software analyst	Design case report forms; programme electronic data capture systems; undertake system testing; data cleaning activities; reports and dataset preparation
Study site monitors	Conduct source data verification; confirm site records are being maintained appropriately; training study site staff
Study administrators	Assist with Trial Master File maintenance; manage daily communications

^a^Note: tasks may be allocated to different roles or disciplines, depending on available staff

## Staff supervision

Co-applicants sign documents to indicate they are taking responsibility for document content. The lead investigator and senior statistician are commonly asked to sign considerable trial-related documentation, including process documents that are not submitted to ethics committees. They may omit to fully review and consider the contents before signing, if they assume the person drafting the documents is sufficiently expert that only a cursory review is required, particularly if they perceive that the document content will not significantly impact trial quality.

Operational staff can feel unsupported if key trial documents are ambiguous or, conversely, feel undervalued and become resentful over the course of multiple trials if they are compensating for expertise gaps in the co-applicant team, without due recognition. This increases the chance of staff loss. In a study of factors influencing staff retention, the ‘supervision’ variable had the highest mean score with relation to turnover intention [4]. Investigator leadership is identified as an important influencer of clinical research associate job satisfaction and retention [5]. Project managers leave due to dissatisfaction with their supervisors and project management turnover directly affects the project team, negatively disrupting project performance [6].

It has been suggested that ‘*the most intellectually challenging part of a clinical trial, the part that determines success or failure, is the part between protocol development and data analysis*’ [7] and that ‘*only the most intelligent, industrious, and imaginative clinical trial managers can pull off big trials—the ones that change clinical practice*’ [7]. The success of a clinical trial should not rest solely on the shoulders of the operational staff employed on a grant. Nor should it rest on the luck of an investigator in managing to recruit the most intelligent, industrious and imaginative clinical trial manager or, indeed, other operational staff. Co-applicants must take time to support the operational staff and understand the operational detail of the study, rather than simply taking a gamble and leaving it ‘in the capable hands’ of the operational staff delegated to support trial conduct.

Trial communications are best structured through regular TMG meetings, typically attended by the lead investigator, trial manager, co-applicant statistician, operational statistician, monitors, data managers and relevant co-applicants, with minutes retained. This group reports to relevant oversight committees, such as the Data Monitoring Committee (DMC) and TSC.

Communications are formalised in standard documentation, including the protocol, used by co-applicants to confirm their intentions to operational staff. As with all communication, it is crucial for co-applicants to ensure they are communicating clearly, clarifying intentions and not overlooking assumptions about comprehension. They must assure themselves that their intentions have been understood.

Standard documentation can act to safeguard the integrity of the trial. What is obvious to co-applicants is not always obvious to operational staff and vice versa. By being clear what co-applicants require to assure trial quality, resolving gaps in shared understanding early and clearly defining what should be escalated to whom when and how, operational staff can ensure responsible co-applicants are presented with relevant information at the appropriate time. With this approach key decisions can be made with appropriate input from both operational staff and co-applicants, minimising the risk of mistaken assumptions arising while ensuring optimal decisions are reached.

More widely, employers must consider how best to ensure career progression is possible for those rare individuals with the qualifications, experience and expertise—i.e. ‘*the most intelligent, industrious, and imaginative*’ [7]—so that they do not leave clinical trials due to lack of career progression. Organisations should recognise that these individuals are operational trials experts and should be invited to join co-applicant teams, or act as named collaborators on grants, once their skills are advanced to a level where they can share responsibility to the funder for trial delivery.

## Five standard documents: assumptions and recommendations

We consider five standard documents that, if developed with a clear and shared understanding, will inform operational staff how to conduct a methodologically robust trial. While a trial will have many other documents, we believe these particular documents require special attention. Relevant co-applicants should be fully engaged in the decisions informing each of these documents, because they carry joint responsibility with the lead investigator, to the funder, for the successful delivery of the trial.

Combined with effective TMG, DMC and TSC meetings, where issues are escalated for discussion in a structured manner, these documents provide clear guidance to operational staff and sites, such that trials can be conducted efficiently and effectively.

These documents and their associated processes should be discussed openly in the context of TMG meetings. They should be reviewed extensively and efficiently and formally approved by the lead investigator and relevant co-applicants. The lead investigator and co-applicant statistician, in particular, must be assured that trial conduct, as specified in these documents, is consistent with their trial design. While operational staff employed on the trial may draft many of these documents, it is reasonable for the trial leadership team to be expected to fully engage in the content and make recommendations for change where needed. Many organisations have processes in place for the sponsor to undertake a formal risk assessment of the trial, the output of which should be incorporated where appropriate into these documents.

### Study protocol

The study protocol is a trial plan containing the co-applicants’ specifications for delivery of trial objectives. This document is often finalised before operational staff are assigned or employed. The development of trial protocol content has been made easier with publication of SPIRIT [8, 9] and TiDIER [10] guidance.

The protocol is a quality control tool [11]. In multicentre trials, in particular, content ambiguity may lead to differing interpretation between co-applicants, operational staff, recruiting sites and oversight committee members. A common issue identified by the authors includes lack of protocol guidance on whether participant data collection should continue if they discontinue the intervention. In early phase pharmaceutical trials, it is not unusual for the protocol to instruct that participant’s data collection cease if they discontinue intervention. Operational staff and study sites, experienced in such trials, may assume in good faith that data collection in late-phase academic-led trials should also cease in such circumstances, unless there is clear and unambiguous protocol guidance to the contrary. This mistaken assumption can lead to poor follow-up data or randomised ‘non-completers’ being omitted from the trial dataset entirely.

The setup phase of a trial is hectic, but a few hours spent verifying protocol interpretation is invaluable. In an early TMG meeting, the protocol should be reviewed, section by section, to bring to the surface any assumptions that may be held and ensure clarity on content. Before the meeting, attendees should thoroughly review the protocol against the SPIRIT [8, 9] and TIDIER [10] guidance and identify points for clarification. An explicit discussion about protocol non-compliance that requires escalation to the TMG is recommended. Ambiguities, omissions or errors should be rectified via a protocol amendment. Verbal clarifications are not recommended since they may not be communicated to recruiting sites or may be forgotten over time, particularly in the event of staff turnover.

Trial protocols will also be discussed and agreed at the first meeting of the TSC, usually a joint meeting with the DMC conducted before participant recruitment commencing.

### Case report form (CRF)

A CRF is a protocol driven document used to standardise trial data collection. It is used by recruiting sites to record data and by database developers for system specification. A CRF must be comprehensive and user-friendly since upon the completion of all trial activities, the ‘product’ of the trial is the final dataset which forms the basis of the analysis and primary publication.

Validated measures are often sourced from previous studies and are assumed to contain no errors. This is not a safe assumption as validated measures are commonly re-typed from paper sources, introducing errors, or consciously adapted for use in prior studies. Statisticians may incorrectly assume the measure used is the original validated version. Such measures should be sourced from the authors or distributors. The scoring algorithm should be available to the statisticians before the trial begins.

Given the time, money and effort to deliver a trial, co-applicants using the data must be intimately involved in developing the CRF and associated database. The risk of incorrect assumptions being made is high and the consequences of misunderstanding significant [12]. Co-applicants must assure themselves the content will permit preparation of DMC, regulatory, ethics and other reports, allow for Consolidated Standard of Reporting Trials (CONSORT) diagram preparation and permit pre-specified primary and secondary analyses. The CRF should be finalised before any data collection begins.

Operational staff should strive to present draft CRFs to the lead investigator and statisticians in a way that permits rapid and detailed review, discussion and amendment. Increased use of web-based electronic data capture systems requires that decisions are made early and with careful consideration, since changing live datasets adds complexity and is best avoided.

As with protocol review, it need not take more than a few hours to carefully review, as a team, each variable on each CRF page to agree wording, format, coding, missing data codes, range checks and validations. Finally, the CRF pack should be reviewed alongside the protocol to verify that all planned content is needed; the protocol should then be reviewed alongside the CRF pack to verify from the opposite perspective that all requirements are covered in the planned data collection pack.

### Monitoring plan

A trial monitoring plan is a protocol-driven document that details activity required, on site or centrally, to assure compliance with the protocol and relevant regulatory requirements. It contains the specification for monitoring activities undertaken to verify the internal and external validity of the trial. The document may contain instructions in relation to site initiation visits including staff training, verification of data in any electronic data capture (EDC), randomisation and intervention management systems, remote activities conducted between site visits and even scheduling of key trial activities such as reports, budget management and meetings.

Most monitoring tasks are relevant to the validity of the trial. Without co-applicant oversight, time may be spent on monitoring tasks with limited impact on trial quality, at the expense of activities essential to study integrity. Operational staff may omit to communicate important information to co-applicants though lack of awareness of what needs to be escalated, unless the monitoring plan provides proper guidance.

It may seem unlikely to those who have not monitored sites, but even specifying in a monitoring plan that ‘20% of secondary outcome data will be source data verified’ can lead to differing interpretation in respect of what is physically done at site, depending on your underlying assumptions. For example, this may mean all the secondary outcomes of 20% of the patients, 20% of the secondary outcomes of each participant within each visit, the secondary outcomes relating to 20% of the visits an individual patient has over the course of a trial, or the secondary outcomes relating to 20% of the visits patients have completed by the time of the monitoring visit. If multiple staff are undertaking site visits, each may interpret the plan differently.

Instruction to ‘check consent’ or ‘check eligibility’ means different things to different people. One monitor might check only that the source data say the patient consented or that eligibility criteria were met. Another might spend considerable time reading the full historic medical notes to verify eligibility criteria are met. Unless explicit, unambiguous guidance is given, monitors will use their initiative and judgement. At best, this will lead to variation, but, at worst, checks will not be done as the senior project staff expected them to be done. In one trial, a fairly cursory check of recent notes may be adequate. In another, the risks might be much higher and it may be worth spending considerable time reviewing the clinical history. There is no rigid right or wrong. However, sending monitors to site with only a vague notion of what they are meant to do when they get there is not an efficient use of their time.

We recommend the monitoring plan is developed with the active support of relevant co-applicants. The Adaptiertes Monitoring (ADAMON) project [13, 14] explored whether a risk-based approach to study site monitoring was non-inferior to extensive on-site monitoring and concluded that this is the case. A risk assessment document is available (www.adamon.de/ADAMON_EN/Downloads.aspx) which can be used to identify specific risks in the study that on-site or central monitoring aim to mitigate; it should be completed, risks agreed, and strategies to mitigate each specific risk discussed, agreed and documented in the monitoring plan, alongside escalation instructions for each monitoring activity.

The monitoring plan may also include site initiation and greenlight processes, intervention management and distribution processes, central monitoring of EDC system warnings, centralised data checking, pharmacovigilance processes, TMG, DMC and TSC meeting organisation, annual ethics and regulatory reporting, periodic reviews of trial finances, database lock and study close-out processes. Explicit instruction on which EDC system variables should be source data verified, and against which source documents (e.g. paper CRFs, pharmacy logs, medical notes or laboratory results), is recommended. The frequency or timing of each activity should be defined, with guidance on how to select patients or patient visits for review, and escalation parameters agreed with relevant co-applicants relating to each monitoring activity.

The ADAMON approach ensures that the priority focus of monitoring is agreed with relevant co-applicants and that monitors are not taking a ‘one size fits all’ approach. It is an effective way to ensure no incorrect assumptions are made about who is doing what, why, when, where and how, and may include monitoring activity undertaken by multiple individuals. Progress against the plan should be discussed in regular TMG meetings, making it easier for co-applicants to make informed decisions.

A detailed monitoring plan mitigates risk in the event of staff turnover, provides much wanted structure to new monitors and reassures co-applicants that the often-mysterious world of ‘monitoring’ has been thoroughly demystified. Monitoring plan development is time well spent and is as important as protocol and CRF development to the successful conduct of a study.

### DMC report templates

DMC reports are protocol-driven documents presented to the committee overseeing data integrity and patient safety [15]. Report content must be clearly presented to enable the DMC to make recommendations to continue or stop the trial.

Open DMC reports are commonly presented subsequently to the TSC, which usually meets two weeks after the DMC, as the information is relevant to both committees. In some cases, a trial may not need a DMC. However, in these circumstances, the open DMC report can be prepared in the usual way and presented only to the TSC.

Co-applicants and operational staff make assumptions, often based on how previous trial teams have worked, as to what activities are undertaken by which staff. When team members come to the trial with prior expectations and assumptions of roles, it may be unclear what data sources are to be used for different aspects of the DMC reports and who should prepare tables or CONSORT diagrams. In practice, this can lead to inaccurate data being presented to the DMC, either due to the use of ‘informal’ data sources which contain estimates rather than raw data (e.g. tracking spreadsheets) or due to errors in data manipulation by non-statisticians (e.g. trial managers or data managers creating CONSORT diagrams).

The DMC charter and CONSORT diagrams may be prepared by different staff in different teams and agreement should be reached, based on relative skills and experience, on who will draft and circulate these documents. In most trials, the operational statistician drafts DMC report templates, the co-applicant statistician and lead investigator review them and the DMC members approve or request changes [16].

We recommend that a content review of the DMC charter and blank DMC template reports are scheduled at an early TMG, as is done with the protocol, CRF and monitoring plan, to ensure the operational staff understand what is being reported and are clear what data inform the reports.

The TMG should agree what data the statistician requires for DMC reporting and the data cut-off points and the timing of related monitoring activities for each data source.

Consideration should be given to verifying that serious adverse event (SAE) reports are entered in the EDC system before DMC report preparation, not just faxed or emailed to the coordinating centre, or they may be omitted from reports. A mechanism to communicate emergency code breaks to the statisticians should be agreed.

Agreement should be reached upon what data top line CONSORT reporting will be based and how that will be communicated to the statistician. Individual patient level data, including screen failure data, can only be entered in the trial EDC system once a participant has consented to screening. However, if the top line of CONSORT will include a count of the overall numbers of potentially eligible participants within the site, including those who were not approached or who declined to participate, consideration will need to be given to how this data will be collected, collated and communicated as aggregate data to the trial statistician.

### Statistical analysis plan (SAP)

The SAP is a key document involved in the transparent reporting of clinical trial data. A SAP contains a more technical and detailed elaboration of the principal features of the analysis described in the protocol and includes detailed procedures for executing the statistical analysis of the primary and secondary variables and other data [17]. A comprehensive template for constructing a minimum set of items for inclusion in a SAP is available [18].

The meaning of the term ‘visit window’ can differ between staff within the trial, leading to data being wrongly omitted from the dataset. Different staff may make assumptions about the purpose of visit windows, the validity of any data collected outside visit windows and the relative importance of visit windows around particular study visits such as the primary outcome visit. Trial databases can, technically, be programmed to reject data outside visit windows and if the operational staff believe data to be ‘invalid’ if collected outside visit windows, this may be programmed into the database system without the knowledge of the trial statisticians.

The trial statisticians may assume other operational staff know what is important to communicate to them or that no issues are arising. The trial manager or monitor may assume the statistician does not need to know about a particular issue or already knows by some other mechanism.

We recommend that the co-applicant or operational statistician present the SAP in the context of a TMG, in order that any mistaken assumptions that the statisticians, trial manager or other operational staff may hold about the trial conduct are identified early, when it is still possible to prevent issues.

A review of the SAP in the context of a TMG provides an opportunity to review how issues relevant to DMC report preparation or analysis should be communicated to the statistician. Examples of important issues include situations where patients cross trial arms unintentionally, emergency code breaks or accidental unblinding occur, specific cases where primary outcome integrity might be compromised or serious breaches of Good Clinical Practice (GCP) are identified that may be crucial to the analysis. While these should have been addressed in the protocol, CRF, monitoring plan or DMC report development stages, a SAP review is the final opportunity to identify any areas of concern.

## Discussion

A healthy philosophical approach to take when developing the five standard documents discussed above is embodied in Saint-Exupery’s Law, ‘*Perfection is achieved, not when there is nothing more to add, but when there is nothing left to take away*’ [19].

Staff at all levels must strive to build working relationships with colleagues in the trial team conductive to open discussion and cross-team learning, in order to avoid erroneous assumptions harming the trial.

Writing intentions clearly within the five standard documents can be difficult. There may be a temptation to be ‘vague’ in the mistaken belief that being specific can introduce rigidity or inflexibility. Clarity does require careful thinking at an earlier stage, but this is to be encouraged. Sites and operational staff reading between the lines when documents are unclear poses a far greater risk to trial conduct than any benefits vagueness can offer.

Tunnel vision about each co-applicant or operational staff members ‘remit’ and preconceptions about role-task definitions, with poorly supervised delegation of tasks that require methodological or clinical oversight—i.e. issues that may impact the validity of the trial or patient safety—harms trials and may even constitute a breach of GCP. Activities that require co-applicant oversight are not just ‘operational details’ and, if perceived as such, can be inadvertently neglected due to competing academic pressures, gaps in the skill mix of the co-applicant team or excessive reliance on the expertise of inexperienced operational staff. Organisations may wish to include specific training in delegation and supervision arrangements, to ensure investigators are aware that inappropriate delegation or inadequate supervision of those delegated constitutes a breach of GCP.

Task delegation should be considered by the lead investigator during team formation to ensure a well-rounded team is available to support operational staff. Investigators should consider inviting senior operational staff—the ‘*most intelligent, industrious, and imaginative clinical trial managers’* [7]—to be co-applicants or named collaborators, to complement the knowledge and skills of other co-applicants, rather than just hope they will attract grant funded operational staff who are exceptional. Steps should be taken to ensure organisations appreciate the need to retain experienced operational staff between trials so that expertise developed is not lost.

In trials where creative solutions to regulatory requirements are needed (e.g. in large pragmatic trials), specialist knowledge of electronic data capture systems is required (e.g. in multi-centre trials with multiple follow up visits) or the medication supply is particularly complex (e.g. multiple manufacturing runs required and issues of blinding relating to distribution to sites), skilled senior staff with the required expertise should be invited to join co-applicant teams or act as named collaborators.

In an increasingly complex clinical trials environment, those working in trials must strive to bring to the surface assumptions that might cause harm to their trial. A significant challenge for the lead investigator is recognising mistaken assumptions that are being made within the team, both in terms of the trial methodology and task delegation. Careful preparation of the five standard documents, with engagement from all team members, will mitigate the risk of such assumptions.

## Data Availability

Not applicable

## Abbreviations

ADAMON: Adaptiertes Monitoring
CONSORT: Consolidated Standards of Reporting Trials
CRF: Case report form
DMC: Data Monitoring Committee
EDC: Electronic Data Capture
SAE: Serious adverse event
SAP: Statistical analysis plan
TMG: Trial Management Group
